# Bacterial Load in Daily Urine Samples of Patients Infected with* Mycoplasma genitalium*, Mutation Analysis, and Response to Treatment

**DOI:** 10.1155/2016/8382469

**Published:** 2016-10-18

**Authors:** M. Gossé, S. A. Nordbø, B. Pukstad

**Affiliations:** ^1^Department of Cancer Research and Molecular Medicine, Norwegian University of Science and Technology, Trondheim, Norway; ^2^Department of Laboratory Medicine, Children's and Women's Health, Norwegian University of Science and Technology, Trondheim, Norway; ^3^Department of Medical Microbiology, St. Olavs Hospital, Trondheim University Hospital, Trondheim, Norway; ^4^Department of Dermatology, St. Olavs Hospital, Trondheim University Hospital, Trondheim, Norway

## Abstract

*Objective*. Increasing macrolide resistant strains of* Mycoplasma genitalium* is a challenge, and to differentiate between treatment failure and reinfection a timely test of cure (TOC) is warranted. The aim of this study was to evaluate the best time for TOC after five days' treatment of* Mycoplasma genitalium* infection with azithromycin.* Methods*. Nineteen patients with positive PCR for* Mycoplasma genitalium* in urine provided urine samples daily for 2 weeks and on days 21, 28, and 35. Samples were tested by a commercial qPCR and by sequencing of the 23S rRNA gene.* Results*. Eight patients with a wild type of* Mycoplasma genitalium* responded successfully within four days after treatment initiation. Eleven patients had a mutation in the 23S rRNA gene. These samples exhibited high variations in bacterial load, and some patients tested negative at several time points during the observation period.* Conclusions*. Day-to-day fluctuations in the mutation samples allow for false negative TOC during the first 5 weeks after start of treatment. Due to increasing macrolide resistance of* Mycoplasma genitalium*, pretreatment mutation analysis is recommended. When a wild type is verified, TOC performed one week after initiation of treatment is suggested.

## 1. Introduction

*Mycoplasma genitalium* is a sexually transmitted bacterium able to cause acute and chronic nongonococcal urethritis (NGU) in men [1, 2] and urethritis, cervicitis, and pelvic inflammatory disease (PID) in women [1–3], although in many cases the infection is asymptomatic [4, 5]. The recommended treatment for NGU when caused by* Chlamydia trachomatis*, or when bacterial species is unknown, is a 7-day course of doxycycline (100 mg twice daily) [6]. Studies show that doxycycline is less effective in treating* M. genitalium*, with varying cure rates across studies and a pooled cure rate of 42% [7]. 1 g of azithromycin given as a single dose has shown higher cure rates [8, 9], but an extended course of 1,5 g given as 500 mg on day 1 and 250 mg on days 2–5 is recommended in Scandinavia to avoid induced macrolide resistance [10, 11]. Unemo et al. showed that this treatment regimen is also effective in eradicating* C. trachomatis*. In recent years, several studies have shown an increase in treatment failure following the use of azithromycin in* M. genitalium *infections [12–14]. Jensen et al. found mutations in region V of the 23S rRNA gene in positions 2058 and 2059 (*Escherichia coli *numbering) accounting for some of the macrolide failures seen [15]. High bacterial load may also be associated with treatment failure [16, 17]. In cases of treatment failure moxifloxacin, a fluoroquinolone, is recommended as a second line treatment [7].

The recommended time for test of cure (TOC) in Norway is 4-5 weeks after treatment initiation (Norwegian Institute of Public Health). Most countries have a similar practice [6]. To our knowledge, there are very few published studies evaluating the optimal time for TOC for* M. genitalium. *In a study by Bissessor et al., samples taken at day 14 yielded the same result as samples taken at day 28 after treatment, and the authors propose TOC to be conducted after 14 days [14]. Reducing TOC to 14 days would reduce false positive results due to reinfection and enable initiation of second line treatment at an earlier time, thus reducing potential transmission of resistant strains. A recent study by Falk et al. states, however, that a TOC should not be conducted earlier than 3-4 weeks, due to induced resistance in a limited number of samples [18].

The aim of this study was to evaluate the best time for test of cure after treating* M. genitalium* infection with a five-day course of azithromycin. Response to treatment was monitored over a period of 5 weeks in nineteen patients with positive PCR for* M. genitalium* in urine. The samples were analyzed and compared with mutation analyses and outcome of treatment. Both patients with symptoms and without symptoms were included.

## 2. Materials and Methods

### 2.1. Patient Recruitment and Sample Collection

Recruitment took place at the Outpatient Clinic of Venereal Disease, St. Olavs Hospital, Norway, and at a sexual health clinic for students. Untreated patients testing positive for* M. genitalium* were asked to participate in the study. Those testing positive for* Chlamydia trachomatis *or* Neisseria gonorrhoeae* were not included. Upon recruitment, participants signed a consent form and provided a pretreatment urine sample. To avoid spread of infection in case of treatment failure and reinfection during the study period, the participants were instructed to use a condom until test of cure results were available. Twenty patients participated in the study, six males and fourteen females, and the median age was 22 years (range 18–33 years). Four patients presented with self-reported symptoms of discharge, in one case bloody, pruritus, and dysuria, whereas the others were asymptomatic throughout the study period. All patients received a five-day azithromycin 1,5 g extended course: 500 mg on day 0 and 250 mg on days 1–4 (project day numbering). Patients were provided with all necessary equipment and an instruction manual. On day 1 through day 14, all patients took a first void urine (FVU) sample daily and transferred it into 9 mL plastic tubes. The patients were instructed to provide the first urine of the day or at least wait for one hour after micturition before sampling, to allow potential bacteria to accumulate in the urethra. The tubes were then stored at −20°C in the participants' freezers. The same procedure was conducted on days 21 (three weeks), 28 (four weeks), and 35 (five weeks). At the end of the sampling period, the seventeen urine samples were transferred to the laboratory and stored at −80°C. The urine samples from all patients were then DNA extracted on the same day, in a total of 15 batches, and the eluates were stored at –80°C until analyzed. Collectively this resulted in the possibility of two freeze-thaw cycles for the urine samples and one for the eluate. Patient recruitment and data collection commenced in October 2014, and the last set of samples were collected in June 2015.

One patient (patient 5) decided to withdraw from the project, and patient 21 was therefore recruited. Another patient (patient 11) was later excluded because it turned out she had been included based on a positive vaginal swab sample and not urine. She did have six positive urine samples throughout the study period, but as the first couple of samples were negative in urine she did not fulfil the inclusion criteria. The total number of participants in the project was therefore nineteen.

### 2.2. DNA Extraction

To obtain maximal sensitivity, DNA was extracted from 1 mL urine using the NucliSens EasyMag system (bioMérieux SA, Marcy l'Etoile, France), yielding 55 *μ*L of eluate. The eluates were stored at −80°C until further processing.

### 2.3. Quantitative PCR

Quantitative PCR (qPCR) was conducted using a CFX96 Real-Time PCR instrument (Bio-Rad Laboratories Inc., Hercules, CA, USA) and the FTD Urethritis basic detection kit (Fast-Track Diagnostics Ltd., Sliema, Malta), for the simultaneous detection of* Mycoplasma genitalium*,* Chlamydia trachomatis,* and* Neisseria gonorrhoeae*. All qPCR runs contained an internal positive control to ensure that no inhibitors were present in the DNA extracts. Quantitation of bacteria per mL was performed according to the manufacturers' instructions. A validated equation was used to calculate copy numbers per mL. All samples belonging to the same patient were tested in the same run.

### 2.4. Sequencing

A minimum of one sample from each patient was sequenced using Beckman Coulter CEQ8800 Genetic Analysis System (Beckman Coulter Inc., Brea, CA, USA). In an attempt to secure a solid sequencing result, the samples with the lowest cycle threshold values were chosen for sequencing. We used Rapid Cleanup Enzyme Set (New England BioLabs Inc., Ipswich, England) for PCR product cleanup and Bio-Analyzer (Agilent Technologies Inc., Santa Clara, CA, USA) for DNA concentration measuring. GenomeLab™ DTCS Quick Start Kit (Beckman Coulter Inc.) was used for dye terminator cycle sequencing of a 241 bp amplicon from the 23S rRNA gene (sense primer “5-GGTTAAAGAAGGAGGTTAGCAATTT-3” and anti-sense primer “5-AGCTACAGTAAAGCTTCACTGGG-3”) (TIB Molbiol, Berlin, Germany).

### 2.5. Ethics and Biobanking

The study was approved by the Regional Ethics Committee (REK Midt-Norge). All urine samples and DNA eluates were registered at the Regional Biobank of Central Norway and stored at −80°C.

## 3. Results

Ten of the nineteen patients tested positive for* M. genitalium* and nine tested negative at the end of the follow-up period (Table 1). New treatment was considered among those still testing positive at this time. Eight patients received moxifloxacin immediately after the positive test result from the TOC was available. Two patients (3 and 6) received a second course of azithromycin because reinfection could not be excluded. Patient 3 received moxifloxacin at the next appointment whereas patient 6 never showed up for a new appointment. Samples from one of the patients testing negative at TOC (patient 16) later revealed a mutant strain, whereas the rest of the patients testing negative at TOC were infected with a wild type. One patient (patient 20) was unable to provide a pretreatment sample because he had just emptied his bladder. Another patient (patient 3) had a negative pretreatment sample, though all the following posttreatment samples were positive, and the reason this patient was recruited was because he had a positive test ten days prior to start of sampling.

### 3.1. Mutation Analysis

**Figure 1:**
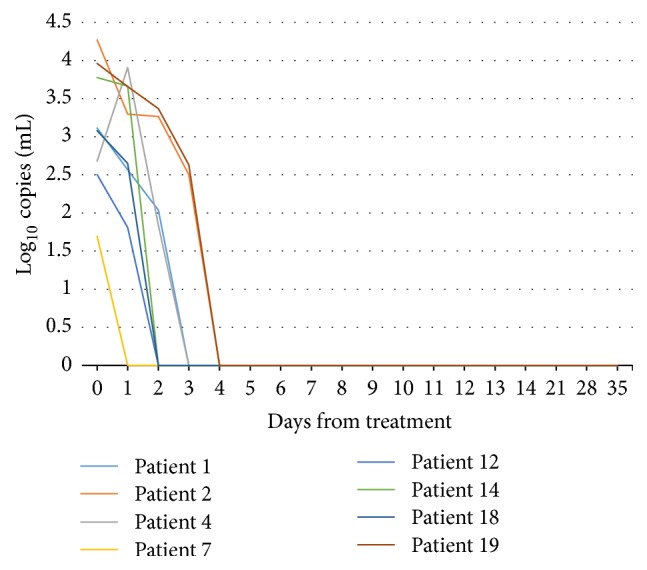
Bacterial load in participants infected with a wild type strain. Day 0 is pretreatment. All participants had negative urine samples from day 4 after treatment.

Sequencing showed that eight patients had a wild type strain (Table 1). Patient 7 was negative on day 1; patients 12, 14, and 18 were negative on day 2; patients 1 and 4 were negative on day 3; patients 2 and 19 were negative on day 4 (Figure 1). The eleven patients who experienced persistent infection had mutations in position 2058 or 2059. Four patients had an A2058G mutation and seven had an A2059G mutation. Among the patients with an A2059G mutation, one (patient 16) had positive samples the first fourteen days but had a negative PCR on days 21, 28, and 35 and at TOC.

### 3.2. Mutant Types Stayed Positive throughout Most of the Test Period

**Figure 2:**
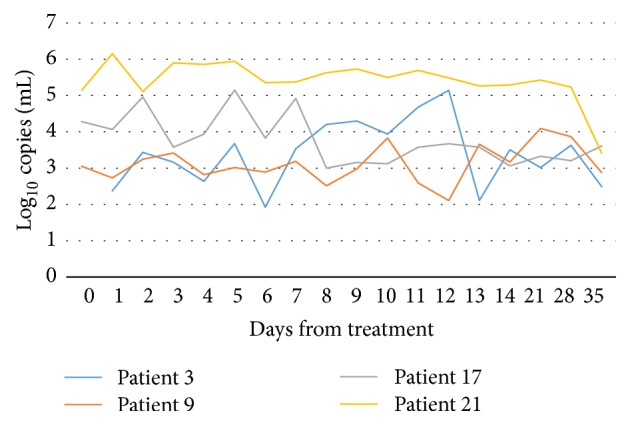
Bacterial load in participants infected with an A2058G mutation. All samples stayed positive throughout the whole test period.

**Figure 3:**
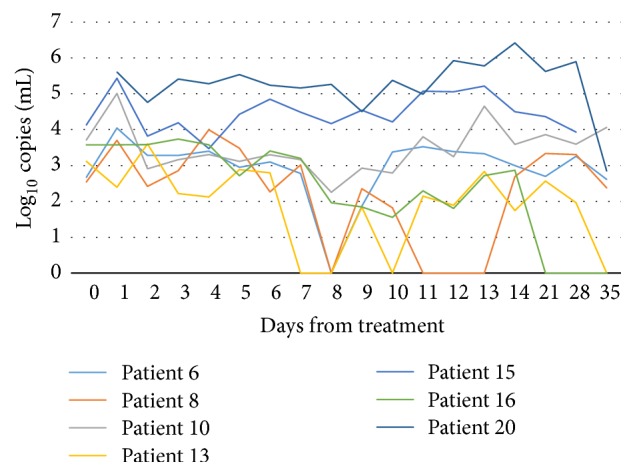
Bacterial load in participants infected with an A2059G mutation. Some patients had sporadic negative samples during the test period.

Patients 3, 9, 10, 15, 17, 20, and 21 had positive urine samples all throughout the test period, though the bacterial load fluctuations varied considerably from day to day (Figure 2). Patients 6, 8, 13, and 16 had some negative samples during the test period (Figure 3). These were all infected with an A2059G mutant type. Patient 6 had a negative sample on day 8, but from day 9 and forward all samples were positive. Patient 8 had negative samples on day 8 and days 10–13, and patient 13 had negative samples on days 7, 8, 10, and 35. TOC taken on day 38 was positive, however, and the patient was treated with moxifloxacin with good effect. Patient 15 failed to deliver a test on day 35, but the test of cure on day 37 was positive.

### 3.3. Symptoms

Four patients (patients 9, 13, 15, and 17), three women and one man, presented with symptoms and were all infected with a mutant type. Patients 9 and 17 had an A2058G mutation and patients 13 and 15 had an A2059G mutation. Patients 15 and 17 experienced complete relief of symptoms after treatment; patient 9 experienced a lasting reduction but not complete relief; patient 13 experienced a reduction of symptoms but a flourishing of symptoms around day ten. All symptomatic patients received moxifloxacin after first TOC and delivered urine with negative PCR for* M. genitalium* at their next TOC.

## 4. Discussion

At present, the IUSTI European guideline recommends performing TOC after infection with* M. genitalium* 3–5 weeks after initiation of treatment [6]. Patients are instructed to use a condom while waiting for the results of their TOC, but adherence is variable. Thus, there is a risk of reinfection during this period and also spread of mutant strains if treatment has not been successful. Being able to conduct a TOC as early as possible is therefore warranted. In this study we followed nineteen patients with* M. genitalium* detected in urine closely for 5 weeks. They were all treated with the standard five-day regime of azithromycin. There was a clear pattern between wild type strains, where treatment was 100% effective during the 5 weeks of follow-up, and mutants, where the treatment had little or no effect. Patients infected with the wild type strain all delivered negative urine samples within four days of treatment.

### 4.1. Persistent Infection in Most Patients with Mutant Strains

Most patients with mutant strains stayed positive throughout the test period, but there were day-to-day fluctuations and even some negative samples. Even though patients were instructed to sample first void urine from the first toilet visit of the day or make sure not to micturate within at least one hour before sampling, the variations in bacterial load might be due to variations in time points of micturition and how long urine had stayed in the bladder before micturition. As far as we know, no one has conducted a study assessing the appropriate time for testing of* M. genitalium* after last micturition, and thus the impact of this is not known regarding bacterial load. The patients stored the samples in their own freezers and brought them to the clinic. Cyclic changes in freezer temperature and the possibility of thawing upon transport might have influenced the quantitative results to some degree.

The patients who had mostly positive but some negative samples during the test period were all infected with an A2059G mutant type. Several of the samples taken on adjacent days showed a bacterial count of <100 copies/mL, indicating that the samples might be false negative, due to a low bacterial load. Patient 16, who had positive samples the first fourteen days but had negative samples on days 21, 28, and 35, had an A2059G mutation. The first week the average bacterial load was 3160 copies/mL (range 514–5460 copies/mL) and the second week the average load was 245 copies/mL (range 36–736 copies/mL). Even if there were day-to-day variations, there was a definite decline. However, it is not possible to conclude whether this was due to an effect of azithromycin or not. An additional sample taken on day 47 was also negative, indicating that the patient's immune system may have eradicated the infection on its own. No additional treatment was given to this patient, as the infection was cleared.

The target of the PCR used for the detection of* M. genitalium* is the mgpB gene. Sequence changes in this gene due to mutations and recombinations frequently occur, even within a time period as short as 10 days. Hence, mismatches between primers, probe, and the target may give rise to weak signals and even false negative results [19, 20]. The rate of spontaneous clearance of* M. genitalium* is not well documented, although two cohort studies have examined aspects of its natural history. Oakeshott et al. reported that only 26% of* M. genitalium* positive women remained positive after 12–21 months [21], and Vandepitte et al. reported a 93% spontaneous clearance rate after 12 months [22]. As pointed out by Smieszek et al., investigating the natural history of a sexually transmitted pathogen is difficult due to risk of reinfection during the follow-up [23].

All four symptomatic patients happened to be infected with a mutant type, and all four symptomatic patients reported some relief of symptoms after treatment. Azithromycin treatment has been reported to possess anti-inflammatory properties, but its mechanism of action is still not fully understood [24]. In our study, the bacterial load did not differ substantially between symptomatic and nonsymptomatic patients.

### 4.2. Time for Test of Cure

In our limited material almost all patients had matching results on day 4 and day 35. This suggests that a TOC could be performed within a week, giving the benefits of early testing. Though induced resistance did not occur in our material during the 5 weeks of follow-up, some researchers have reports of this [15, 18, 25]. Due to such induced resistance it is recommended to perform TOC no earlier than 3-4 weeks after initiation of treatment [26]. Can a negative TOC between week 3 and week 5 after treatment initiation then be trusted? In our study, a positive TOC taken >4 days after treatment indicated a mutant type, but a negative sample taken >4 days after treatment did not exclude the persistence of a mutant strain. These results indicate that a larger study is both important and necessary. One of our patients with a mutant strain (number 13) had a negative result on day 35 and a positive result on day 38. With this in mind, a TOC should be done either very early, for example, one week after initiation of treatment, in order to detect possible mutations early, or after 5 weeks in order to reduce the chance of missing a possible induction of a wild type strain turning into a mutant strain.

In a few cases a negative test can be the result of a sample with a bacterial load below the detection limit of the PCR after macrolide treatment, which might be due to a low-grade infection or due to poor bacterial accumulation because of recent micturition. DNA extraction method, sensitivity of the PCR test, and attention to time of micturition are factors to consider when suspecting false negative results. A negative result can be misinterpreted as successful treatment even if the patient has not been cured.

It is important to note that, in this study, only urine samples were tested and we do not know if the results will apply to swab specimens as well. In the study of Falk et al., no differentiation between urine specimens and vaginal swabs was made [18], but Solberg shows in a recent study that vaginal swab samples are likely to contain a higher amount of bacterial DNA [27]. The superiority of vaginal swabs over urine samples is also shown by Wroblewski et al. [28] and Lillis et al. [29]. A study evaluating time to eradication of* M. genitalium* from vaginal swab specimens compared to urine specimens from women is therefore warranted.

Appropriate TOC after treatment of a mutant strain will depend on treatment given. TOC after quinolone or tetracycline treatment was not evaluated in this study, and further studies regarding this are needed.

### 4.3. High Prevalence of Resistant Types

Macrolide resistant types of* M. genitalium* were detected in 11 (58%) of 19 patients in this study. This is in line with our findings in a larger study [30] in our region and has also been reported by others from Norway [31], England [13], Australia [14], Denmark [32, 33], and the Netherlands [34]. Hence, macrolide resistance is an increasing problem [31, 35–37]. With a high prevalence of resistant types, a mutation analysis should be performed on the first sample, both to avoid treatment failure with azithromycin and to avoid the use of macrolide antibiotics when it is not indicated.

## 5. Conclusions

In this study we found that a five-day azithromycin course is highly effective for eradication of the wild type strain of* M. genitalium* in urine within four days after onset of treatment. Our findings suggest that a TOC already one week after initiation of standard five-day treatment with azithromycin is recommended. However, our study was small, and no firm conclusions can be made. Hence, larger studies, including vaginal and possibly male urethral specimens, are warranted to confirm or discard our results. The prevalence of macrolide resistant strains seems to be increasing in Norway, and pretreatment mutation analysis of* M. genitalium* is recommended to guide treatment.

## Figures and Tables

**Table 1:** Patient number, symptoms, bacterial type, and bacterial load at baseline and on day 4 after treatment with azithromycin 1,5 g. Patients 5 and 11 were excluded from the study. Patient 3 had a negative pretreatment sample but had a titer of 2,36 × 10^2^ copies/mL on day 1. Patient 20 was unable to provide a pretreatment sample due to recent micturition and had a titer of 4,01 × 10^5^ copies/mL on day 1. Patients 13 and 15 had a negative test at day 35 but tested positive at their follow-up appointment a few days later.

Participant number	Symptoms	Bacterial load at baseline, copies/mL	Bacterial load at day 4, copies/mL	Bacterial load at day 35, copies/mL
Wild type				
1	—	1,30 × 10^3^	—	—
2	—	1,86 × 10^4^	—	—
4	—	4,80 × 10^2^	—	—
7	—	4,96 × 10^1^	—	—
12	—	3,18 × 10^2^	—	—
14	—	5,97 × 10^3^	—	—
18	—	1,20 × 10^3^	—	—
19	—	6,97 × 10^2^	—	—
A2058G mutation				
3	—	—	4,31 × 10^2^	3,09 × 10^2^
9	Symptomatic	1,13 × 10^3^	6,64 × 10^2^	7,56 × 10^2^
17	Symptomatic	1,91 × 10^4^	8,66 × 10^3^	4,08 × 10^3^
21	—	1,40 × 10^5^	7,28 × 10^5^	2,56 × 10^3^
A2059G mutation				
6	—	4,70 × 10^2^	2,51 × 10^3^	4,08 × 10^2^
8	—	3,47 × 10^2^	9,92 × 10^3^	2,37 × 10^2^
10	—	5,18 × 10^3^	2,03 × 10^3^	1,16 × 10^4^
13	Symptomatic	1,30 × 10^3^	1,33 × 10^2^	—
15	Symptomatic	1,35 × 10^4^	2,95 × 10^3^	—
16	—	3,79 × 10^3^	3,76 × 10^3^	—
20	—	Not tested on day 0	1,92 × 10^5^	7,06 × 10^2^
